# Strategy for the Management of Macular Edema in Retinal Vein Occlusion: The European VitreoRetinal Society Macular Edema Study

**DOI:** 10.1155/2015/870987

**Published:** 2015-01-29

**Authors:** Ron A. Adelman, Aaron J. Parnes, Silvia Bopp, Ihab Saad Othman, Didier Ducournau

**Affiliations:** ^1^Yale Eye Center, 40 Temple Street No. 3d, New Haven, CT 06510, USA; ^2^Eyecare Medical Group, 53 Sewall Street, Portland, ME 04102, USA; ^3^Augenklinik Universitätsallee Bremen GmbH, Parkallee 301/Universitätsallee, 28213 Bremen, Germany; ^4^Cairo University, Cairo University Road, Oula, Giza, Egypt; ^5^European VitreoRetinal Society, 8 rue Camille Flammarion, 44000 Nantes, France

## Abstract

*Objective*. To compare the efficacy of different therapies in the treatment of macular edema associated with retinal vein occlusion (RVO). *Design*. This is a nonrandomized, multicenter collaborative study. *Participants*. 86 retina specialists from 29 countries provided clinical information, including choice of treatment and outcome, on 2,603 patients with macular edema including 738 cases of RVO. *Methods*. Reported data included the type and number of treatments performed, visual acuities, and other clinical and diagnostic findings. *Main Outcome Measures*. The mean increase in visual acuity and mean number of treatments performed. *Results*. 358 cases of central retinal vein occlusion (CRVO) and 380 cases of branch retinal vein occlusion (BRVO) were included in this investigation. Taking all RVO cases together, pars plana vitrectomy with internal limiting membrane (ILM) peeling alone resulted in an improvement in vision greater than other therapies. Those treated with intravitreal antivascular endothelial growth factor (anti-VEGF) injection alone showed the second greatest improvement in vision. Dexamethasone intravitreal implant alone and intravitreal triamcinolone alone both resulted in modest visual gains. *Conclusions*. In the treatment of macular edema in RVO, vitrectomy with ILM peeling may achieve visual improvement and may be a good option for certain cases. Anti-VEGF injection is the most effective of the nonsurgical treatments.

## 1. Introduction

In population based studies, the age- and sex-standardized prevalence of retinal vein occlusion (RVO) was 5.2 per 1000 with an estimated 16.4 million adults affected by RVO, mostly branch retinal vein occlusion. RVO is the second leading cause of retinal vascular disease after diabetic retinopathy with its prevalence increasing with age, varying with race or ethnicity, and not differing based on gender [1]. Untreated RVO frequently results in vision impairment and significant ocular complications. Macular edema is present in the majority of cases with central retinal vein occlusion (CRVO) and develops in 5–15% of eyes with branch retinal vein occlusion (BRVO) [2, 3].

The treatment of macular edema due to RVO has seen significant changes over the past decade. New treatments and combination therapies continue to emerge with several showing positive results. As directed by the Central and Branch Vein Occlusion Study Groups, for many years macular edema in CRVO was observed, while in BRVO grid laser photocoagulation was applied [4, 5]. Corticosteroids, both intra- and extraocular, have long been used to treat edema with RVO, and the SCORE study results validated this therapy for edema in CRVO while confirming grid laser photocoagulation as superior treatment for edema in BRVO [6, 7]. More recently, treatment with dexamethasone intravitreal implant has shown longer-lasting results in the treatment of this edema [8].

The arrival of antivascular endothelial growth factor (VEGF) agents has not only changed the landscape of treatment of edema in RVO but also reshaped the treatment of most of retinal vascular disease. The use of intravitreal ranibizumab has been extensively studied and is very effective in the treatment of edema due to RVO [9–16]. More recently, intravitreal aflibercept injection for treating macular edema in CRVO has shown promising outcomes [17, 18]. Literature examining combination therapy including anti-VEGF agents is not as abundant. While focal and grid laser photocoagulation is routinely used to supplement anti-VEGF injections, the possible benefit of this combined treatment has not been thoroughly studied. It has been suggested that the addition of grid laser photocoagulation to anti-VEGF therapy to treat edema in BRVO can lead to a better visual outcome and decrease the number of injections needed than if laser was not utilized [19, 20]. A separate prospective investigation found that the addition of a dexamethasone intravitreal implant to anti-VEGF injections also leads to a decrease in the number of injections needed and better vision in the combination group compared to monotherapy [21].

Pars plana vitrectomy (PPV) with peeling of the internal limiting membrane (ILM) has been an option in the treatment of edema in RVO for many years [22, 23]. More recent investigations have shown improvement of edema in both BRVO and CRVO following treatment with PPV [24–27]. The efficacy of combination therapy including vitrectomy is not completely understood. Considering the complexity and cost of comparative prospective studies, it is expected that comprehensive investigations analyzing three or more monotherapies and numerous possible combination treatments for edema in RVO are not feasible. An alternative to such large and prohibitively expensive randomized study with five or more treatment arms needs to be evaluated.

European VitreoRetinal Society (EVRS) was founded in 2001 and has over 1,900 retina specialists in its membership [28, 29]. A clinical study was conducted where EVRS members were asked to record information regarding individual cases of macular edema from 2008 to 2011, the treatments performed, and the outcomes attained. 86 retina specialists from 29 countries provided information on 2,603 cases of macular edema over the study period. Each case had at least 6 months follow-up. In this report, we will discuss the treatment and results of those cases with macular edema specifically due to RVO.

## 2. Methods

The EVRS macular edema study was a nonrandomized, multicenter study, which analyzed the treatment outcomes following different therapeutic interventions and treatment combinations for each etiology resulting in macular edema. This paper focuses on cases of edema due to RVO and their treatment outcomes.

During the reporting period from 2008 to 2011, participating EVRS members entered information regarding individual cases of macular edema. The clinical details for each case were entered into an online questionnaire on the EVRS website. Eighty-six retina specialists reported 2,603 cases of macular edema with 6 months to 2 years of follow-up at the conclusion of the study. The results were analyzed by the French INSEE (National Institute of Statistics and Economic Studies).

The clinical details reported from each case included the type and number of treatment(s) utilized, the pre- and posttreatment visual acuities, the specific dates of treatments and visual assessments, and lens status. Macular optical coherence tomography (OCT) measurements were not reported by the surgeons in this investigation. Any complications were also reported including increased or new cataract, increased intraocular pressure, retinal detachment, vitreous hemorrhage, choroidal detachment, and macular hole. After having cleaned the database, the global working sheet was sent to each contributor, masking the name of the other contributors, so that cleaning accuracy could be agreed upon.

This study was conducted in 29 countries and the regulations and Institutional Review Board requirements varied at each location. Given this, every physician involved in the study was responsible for following the rules and regulations of each individual country and institution. Also, the design and ethical aspects of the study have been approved by the EVRS Ethics Committee.

The study encountered a few problems. Following stratification of the macular edema etiologies, a smaller number of subgroups in the RVO cohort limited the ability to achieve statistical significance for each of them. There were 358 cases of macular edema associated with CRVO and 380 cases associated with BRVO. As there is no universal standard in management of macular edema in RVO, treatment complexity and the use of numerous combination therapies in each subgroup hindered a meaningful comparison due to further division of the subgroups to monotherapy and treatment combinations cohorts. To overcome these limitations and difficulties, the results are presented as trend lines displaying change in visual acuity over time to compare treatment outcomes. The trend lines represent data points plotted according to a mean number of lines of vision improvement (in LogMAR) at a specific follow-up visit (in months from initial treatment). Second order polynomial regression trend lines were used, as they best illustrate the effect of treatment in this complex setting. To limit error, only data points averaged from three or more cases were included in the analysis. Finally, a separate analysis was done to display final visual improvement according to pretreatment visual acuity. Trend lines are useful in comparing therapeutic groups. A trend line combining the results for all treatments of RVO was compared to plotted results for individual treatments and their average pretreatment visual acuities.

## 3. Results

Of the 2,603 cases of macular edema presented, four etiologies of macular edema had numbers large enough to study totaling 2,159 patients. 358 were CRVO, 380 were BRVO, 870 were diabetic macular edema, and 551 were epiretinal membranes. The focus of this paper is cases of CRVO and BRVO and the baseline demographic patient data is displayed in Tables 1 and 2, respectively. The baseline demographic patient data for all RVO combined is displayed in Table 3.

**Figure 1:**
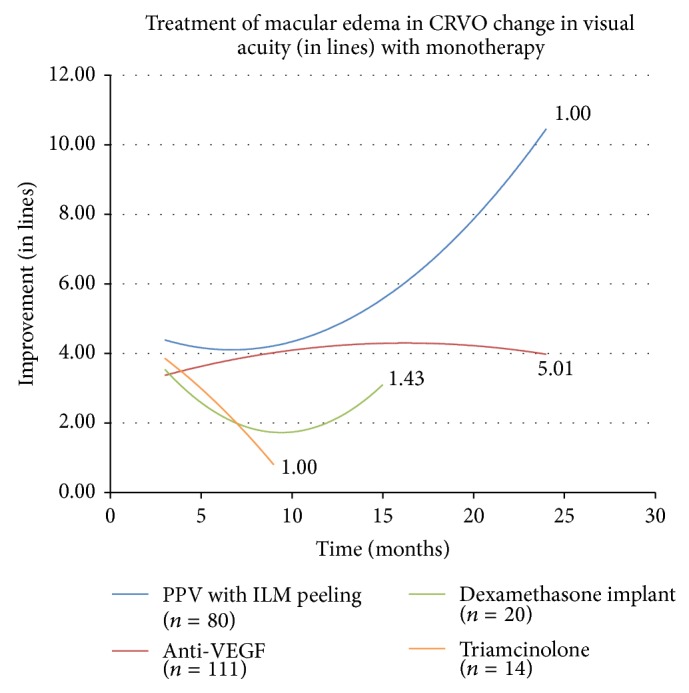
CRVO is central retinal vein occlusion, PPV is pars plana vitrectomy, ILM is internal limiting membrane, and VEGF is vascular endothelial growth factor.

**Figure 2:**
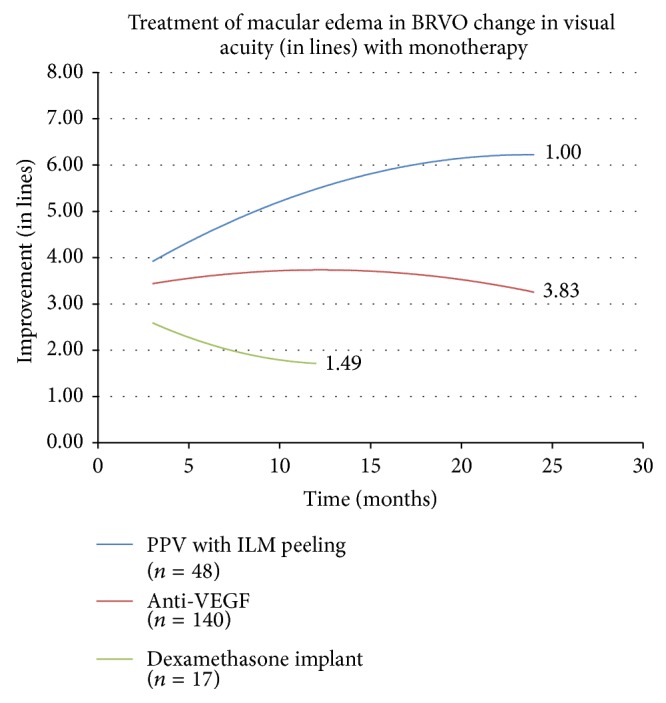
BRVO is branch retinal vein occlusion, PPV is pars plana vitrectomy, ILM is internal limiting membrane, and VEGF is vascular endothelial growth factor.

The initial investigation analyzed the effects of monotherapy on edema in RVO and compared their efficacy. The change in visual acuity over time in response to each monotherapy on edema in CRVO and BRVO is displayed as separate second order polynomial regression trend lines in Figures 1 and 2, respectively. The trend lines for anti-VEGF therapy can be followed out to 24 months where treatment leads to an improvement of 3.958 lines of vision on the LogMAR chart in CRVO and 3.2415 lines in BRVO. The trend lines for PPV with ILM peeling displayed improvement in visual acuity at 24 months with a gain higher than other therapies. The trend lines for dexamethasone intravitreal implant and triamcinolone monotherapy, which are truncated due to the fact that fewer than three cases were reported at the later follow-up periods, showed a modest improvement. The numbers adjacent to the trend lines indicate mean number of treatments for each therapeutic intervention.

**Figure 3:**
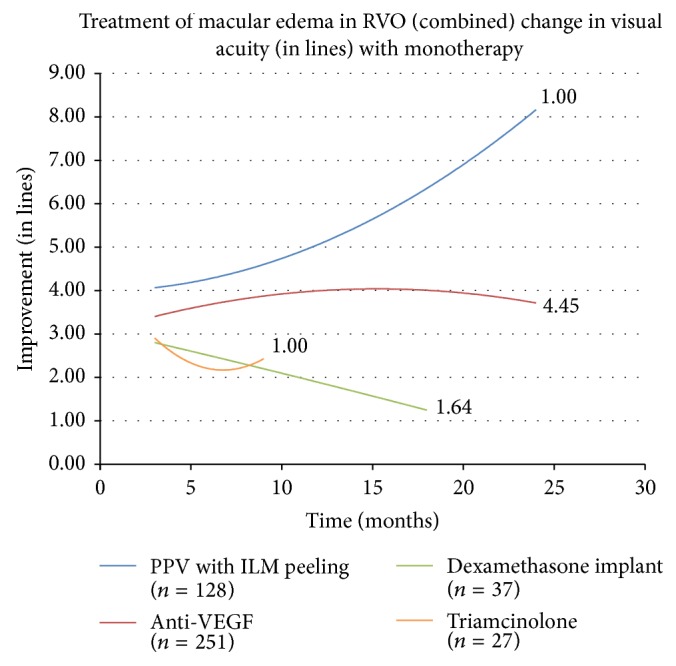
RVO is retinal vein occlusion, PPV is pars plana vitrectomy, ILM is internal limiting membrane, and VEGF is vascular endothelial growth factor.

The effect of each monotherapy on all cases of RVO combined was then analyzed and the trend lines are displayed in Figure 3. The trend line for anti-VEGF therapy can be followed out to 24 months where treatment leads to an improvement of 3.740 lines. The trend line for PPV with ILM peeling again displayed the best improvement in visual acuity. The trend line for dexamethasone intravitreal implant, which again is truncated due to a lack of cases reported at later follow-up times, showed a modest improvement. Also for all RVO, treatment with intravitreal triamcinolone monotherapy showed a modest gain at 9 months.

**Figure 4:**
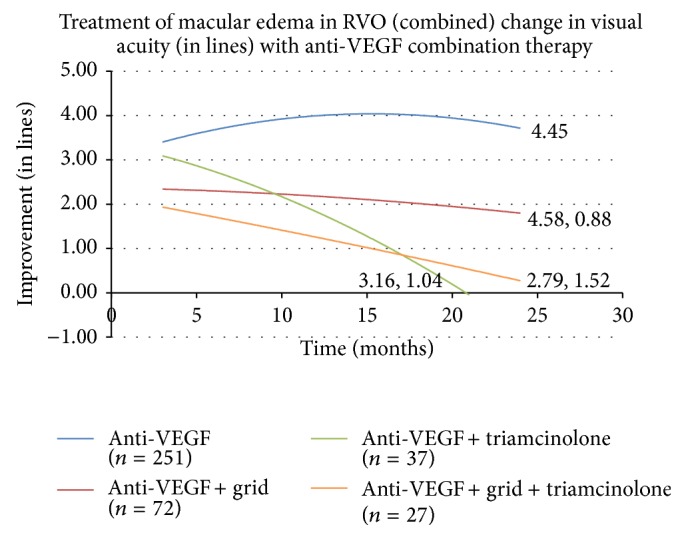
RVO is retinal vein occlusion and VEGF is vascular endothelial growth factor.

Next, combination therapy with anti-VEGF agents was evaluated and compared in all RVO combined (Figure 4). The addition of grid laser photocoagulation to anti-VEGF therapy, combination therapy of intravitreal triamcinolone with anti-VEGF treatment, and combination of anti-VEGF therapy, intravitreal triamcinolone, and grid laser were studied. None of these combination therapies matched the gains observed with anti-VEGF treatment alone.

Visual improvement was then evaluated by looking at the percentage of patients achieving greater than or equal to 3 and 6 lines of vision recovery. In this analysis, the final visual acuity reading available was compared to the recorded pretreatment acuity. Monotherapy with either anti-VEGF treatment or PPV with ILM peeling for edema in all cases of RVO was compared using this data (Table 4). 61.7% of the anti-VEGF group and 75.4% of the vitrectomy group gained at least 3 lines of vision. This was a statistically significant difference with vitrectomy showing a better result over anti-VEGF therapy (*P* = 0.008). 26.0% of the anti-VEGF group and 48.4% of the vitrectomy group gained at least 6 lines of vision. PPV with ILM peeling again showed good outcomes (*P* = 10^−5^).

**Figure 5:**
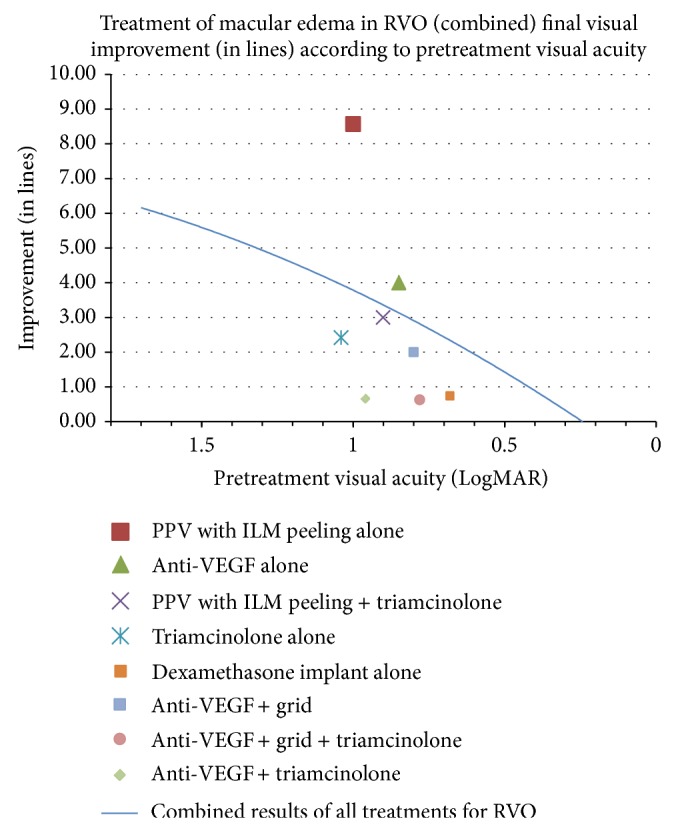
RVO is retinal vein occlusion, PPV is pars plana vitrectomy, ILM is internal limiting membrane, and VEGF is vascular endothelial growth factor.

Another presentation of the data displays treatment outcomes based on final visual improvement according to pretreatment visual acuity (Figure 5). This allows for a comparison of each treatment taking into account the initial visual acuities and the overall combined results for all treatments of RVO seen as a single second order regression trend line. Following the analysis of all possible mono- and combination therapies for edema in RVO, the plotted data points represent the top eight treatments, in terms of visual improvement. PPV with ILM peeling displayed the largest improvement in vision. This therapy was followed by, in order of descending amount of vision gain (LogMAR), anti-VEGF treatment alone, PPV with ILM peeling in combination with triamcinolone, and triamcinolone therapy alone.

## 4. Discussion

New treatments and subsequent combination therapies for macular edema in RVO have provided the present-day retina specialist with choices arguably more ample and complex than those of the previous generation. While these recently suggested treatments are welcome, their role with regard to appropriate first-line and subsequent therapy is not clear. The simple fact that only 11 of the 738 patients with RVO had their edema treated with grid laser photocoagulation monotherapy shows a major shift in treatment philosophy of RVO. This small number did not allow for an analysis and signals a large shift in the international standard of care in recent years. Of course, a large-scale, prospective, and randomized study with treatment arms covering all possible mono- and combination therapies would be ideal to provide an answer. However, such study would likely be very costly and complex to conduct. In this investigation, we present an international nonrandomized multicenter trial evaluating current treatments for edema in RVO. Such study presents the real-life approach of a large number of ophthalmologists from a huge geographical area to the management of RVO.

When monotherapy for edema in RVO was analyzed, treatment with vitrectomy and ILM peeling gave the largest improvement in visual acuity. This held true when cases of CRVO and BRVO were evaluated both separately and in combination. The improvement in vision with vitrectomy was better than other therapies at every time point. Overall, intravitreal anti-VEGF injection was the next most effective solo treatment with a gain of 3.7 lines of vision on the LogMAR chart at 24 months. Less improvement was seen with steroid monotherapy. The addition of grid laser, intravitreal triamcinolone, or both to anti-VEGF treatment did not improve visual outcomes compared to anti-VEGF alone. A traditional analysis showed a statistically significant benefit of vitrectomy over anti-VEGF therapy, in terms of percentage of cases gaining over 3 or 6 lines of vision. The problem with this type of analysis is that it does not take into account pretreatment vision. When initial visual acuity is included in the evaluation and results were adjusted based on initial visual acuity, vitrectomy with ILM peeling is still superior to other treatments and shows over twice the benefit of anti-VEGF injection. The results here suggest that vitrectomy with ILM peeling may provide good long-term benefit in the treatment of edema in RVO.

The significant improvement in edema and vision with vitrectomy seen here is likely the result of a number of factors. Posterior hyaloid removal may contribute to a decrease in edema because of the relief of any tractional component that may be present [30]. Spectral domain OCT has now been used to identify extrafoveal traction that may play a role in edema associated with RVO [31]. Also, improvement in vision may be attained with better preservation of the ellipsoid line and parallelism following PPV. Another possible factor noted in the literature is that vitreous removal may serve to improve oxygenation of the vitreous cavity and retina and prevent photoreceptor loss in RVO [32]. The removal of inflammatory and permeability mediators in the vitreous, including VEGF, may also play a role in improving edema in RVO [33]. A separate mechanism to explain the success of ILM removal involves the healing process it induces at the level of the Müller cells end-feet [34]. This minor injury causes an upregulation of epidermal growth factor receptor (EGF-R) which regulates the healing response. Stem cell proliferation occurs as a result of cell loss and EGF-R stimulates the filling of Müller cells with microfibrils of glial fibrillary acidic proteins (GFAPs) causing a vertical gliosis from the ILM to the external limiting membrane. This process of neural repair has been observed in both the central nervous system following traumatic injury and the retina in the setting of a retinal detachment [35–38].

While the evaluation of outcomes of this study in this manner may be useful, there are inherent limitations and significant disadvantages. Regarding the trend lines, even though several cases were available for each data point, generally fewer cases were available when plotting the data at two-year time point. Although trend lines are useful in comparing efficacy of a variety of treatment groups, they may not be very accurate in measuring the exact amount of improvement. Another limitation is the lack of randomization. However, the current study shows visual outcomes comparable to randomized clinical trials of anti-VEGF therapy for retinal vein occlusions.

The exact treatment parameters for each treatment group were determined by the investigators, leading to multiple treatment groups and smaller numbers to analyze for every mono- or combination therapy applied. In addition, the frequency and order of treatments were not standardized, leaving us with an ability to suggest a treatment, but without exact guidelines for execution. The smaller number of cases receiving each treatment necessitated the grouping of CRVO and BRVO cases together to achieve greater statistical significance. While the pathophysiology of macular edema is somewhat similar in both CRVO and BRVO, they do not necessarily respond exactly to the same treatment [4, 5]. This is a pitfall, however, given other studies that have shown relatively comparable outcomes with the same treatment; it may be reasonable to combine the cases [12, 13].

This study suggests that vitrectomy with ILM peeling may be a good treatment for macular edema due to RVO. The data suggests that vitrectomy may result in improvement in vision in some cases. Intravitreal anti-VEGF therapy is the most effective of the nonsurgical treatments. Future prospective, randomized clinical trials are needed to verify these results and establish a standard of care for the treatment of macular edema in RVO.

## Figures and Tables

**Table 1:** Baseline demographic patient data for CRVO.

	Number of cases	Mean pretreatment Va LogMAR (Snellen)	Standard deviation	Mean number of treatments
Anti-VEGF alone	111	1 (0.1)	0.48	5.01
PPV with ILM peeling alone	80	1.07 (0.09)	0.51	1.00
Dexamethasone implant alone	20	0.77 (0.17)	0.48	1.43
Triamcinolone alone	14	1.23 (0.06)	0.44	1.00

CRVO: central retinal vein occlusion.

VEGF: vascular endothelial growth factor.

PPV: pars plana vitrectomy.

ILM: internal limiting membrane.

**Table 2:** Baseline demographic patient data for BRVO.

	Number of cases	Mean pretreatment Va LogMAR (Snellen)	Standard deviation	Mean number of treatments
Anti-VEGF alone	140	0.72 (0.2)	0.42	3.83
PPV with ILM peeling alone	48	0.87 (0.13)	0.42	1.00
Dexamethasone implant alone	17	0.59 (0.25)	0.27	1.49
Triamcinolone alone	13	0.76 (0.18)	0.38	1.00

BRVO: branch retinal vein occlusion.

VEGF: vascular endothelial growth factor.

PPV: pars plana vitrectomy.

ILM: internal limiting membrane.

**Table 3:** Baseline demographic patient data for all RVO.

		Number of cases	Mean pretreatment Va LogMAR (Snellen)	Standard deviation	Mean number of treatments
Monotherapy	Anti-VEGF alone	251	0.85 (0.14)	0.47	4.45
	PPV with ILM peeling alone	128	1 (0.10)	0.49	1.00
	Dexamethasone implant alone	37	0.68 (0.25)	0.4	1.64
	Triamcinolone alone	27	1.04 (0.09)	0.48	1.00

Combination therapy	Anti-VEGF (1) + grid (2)	72	0.80 (0.16)	0.45	(1) 4.58 (2) 0.88
	Anti-VEGF (1) + triamcinolone (2)	37	0.96 (0.11)	0.49	(1) 3.16 (2) 1.04
	Anti-VEGF (1) + grid (2) + triamcinolone (3)	27	0.78 (0.17)	0.41	(1) 2.79 (2) 1.52 (3) 1.07
	Triamcinolone (1) + PPV with ILM peeling (2)	23	0.90 (0.13)	0.47	(1) 1.15 (2) 1.00

RVO: retinal vein occlusion.

VEGF: vascular endothelial growth factor.

PPV: pars plana vitrectomy.

ILM: internal limiting membrane.

**Table 4:** Final visual improvement for anti-VEGF injection and PPV with ILM peeling monotherapies.

	Anti-VEGF (*n* = 246)	PPV with ILM peeling (*n* = 126)	*P* value
≥3 lines improvement	61.7%	75.4%	0.008
≥6 lines improvement	26.0%	48.4%	10^−5^

VEGF: vascular endothelial growth factor.

PPV: pars plana vitrectomy.

ILM: internal limiting membrane.
